# Self-Help And Recovery guide for Eating Disorders (SHARED): study protocol for a randomized controlled trial

**DOI:** 10.1186/s13063-015-0701-6

**Published:** 2015-04-16

**Authors:** Valentina Cardi, Suman Ambwani, Ross Crosby, Pamela Macdonald, Gill Todd, Jinhong Park, Sara Moss, Ulrike Schmidt, Janet Treasure

**Affiliations:** King’s College London, Institute of Psychiatry, Psychological Medicine, Section of Eating Disorders, The Basement, P059, 103 Denmark Hill, London, SE5 8AF UK; Department of Psychology, Dickinson College, P.O. Box 1773, Carlisle, PA 17013 USA; Neuropsychiatric Research Institute, 700 First Ave. South, Fargo, ND 58103 USA; Department of Psychology, Carleton College, 1 N. College St., Northfield, MN 55057 USA

**Keywords:** anorexia nervosa, eating disorders, guided self-help, peer mentoring, task-sharing

## Abstract

**Background:**

We describe the theoretical rationale and protocol for Self-Help And Recovery guide for Eating Disorders (SHARED), a trial investigating whether a guided self-care intervention (*Recovery* MANTRA) is a useful addition to treatment as usual for individuals with anorexia nervosa. *Recovery* MANTRA*,* a 6-week self-care intervention supplemented by peer mentorship, is a module extension of the Maudsley Model of Treatment for Adults with Anorexia Nervosa and targets the maintenance factors identified by the cognitive-interpersonal model of the illness.

**Methods:**

Patients accessing outpatient services for anorexia nervosa are randomized to either treatment as usual or treatment as usual plus *Recovery* MANTRA. Outcome variables include change in body weight at the end of the intervention (primary) and changes in body weight and eating disorder symptoms at immediate and extended follow-up (6-months; secondary). Change is also assessed for the domains identified by the theoretical model, including motivation, hope, confidence to change, positive mood, cognitive flexibility, therapeutic alliance and social adjustment. Feedback from peer mentors is gathered to understand the impact on their own well-being of providing guidance.

**Discussion:**

Results from this exploratory investigation will determine whether a larger clinical trial is justifiable and feasible for this affordable intervention, which has potential for high reach and scalability.

**Trial registration:**

ClinicalTrials.gov NCT02336841.

## Background

A recent evidence map for research in eating disorders identified a clear need for empirical examinations of interventions for anorexia nervosa [1]. There has also been a recent call to make psychological treatments more widely available by using strategies to make them affordable and scalable, with the possibility of a wide reach [2]. One strategy for meeting these needs involves sharing information and behaviour change skills through guided self-care interventions. This approach has previously demonstrated benefit for people with bulimia nervosa and binge-eating disorder in terms of symptom change [3] and motivation to change [4]. However, there is much variability in terms of effect sizes and the acceptability of these interventions [5]. Although self-care strategies have been less studied for anorexia nervosa, two studies in which they were used as additional steps, either before inpatient admission [6] or after hospital treatment, reported improvements in symptom change and service use [7]. The next step, therefore, involves extending these findings to examine whether or not guided self-care strategies can enhance outpatient care in anorexia nervosa.

We have developed a six-week guided self-help intervention for anorexia nervosa (*Recovery* MANTRA), which consists of a self-care workbook, a series of short video clips, and weekly guidance from peer mentors with personal experience of recovery from an eating disorder. *Recovery* MANTRA is a guided self-care module extension of the Maudsley Model of Treatment for Adults with Anorexia Nervosa (MANTRA), which integrates principles from several related theoretical approaches. These theoretically derived principles are translated into specific behavioural change strategies, such as psychoeducation, specific prompts for instruction and practice and explicit tools for encouragement [8], which participants can use to counteract unhelpful automatic attitudes and behaviours associated with their eating disorder. Preliminary data on the effectiveness of MANTRA (compared with specialized supportive psychotherapy) show that the intervention is rated favourably by participants and associated with a trend for greater benefit (that is, weight gain) in underweight participants (Schmidt *et al.*, unpublished date).

### Theoretical underpinnings for *Recovery* MANTRA

According to the cognitive-interpersonal model of eating disorders [9,10], abnormal eating behaviours in anorexia nervosa are maintained through four factors: (a) positive beliefs about the utility of the illness; (b) a rigid, detail-focused thinking style; (c) avoidance of the experience and expression of emotion; and (d) problematic interpersonal relationships [10]. These elements are thought to develop into habits through a process of neuroadaptation to the illness [11,12]. The *Recovery* MANTRA manual offers a shorter and more focused version of the workbook originally developed for the Maudsley Model of Treatment of Adult Anorexia Nervosa (MANTRA) [13,14]. The *Recovery* MANTRA programme consists of four different intervention modules that target difficulties associated with cognitive processing, emotional functioning, interpersonal style, and poor nutrition. The overall content is aimed at reversing eating disorder habits by making non-eating disorder behaviour more automatic through processes that increase awareness, planning and behavioural experiments [15-17] delivered through a motivational interviewing framework. Moreover, intervention materials are available for delivery in ‘real time’ (as and when desired) through mobile-optimized materials [18], which might enhance the generalizability of learning across new contexts.

It has been suggested that a successful method of enhancing access and availability of psychological interventions is to implement ‘task-shifting’ or ‘task-sharing’ strategies [2]. These involve training nonspecialist individuals (such as peers or non-mental health professionals) to deliver psychological interventions [19]. We have successfully implemented this approach in the treatment of anorexia nervosa by having people with caregiving experience share their skills with other families [20,21]. The positive results from this approach support the use of peer-based guidance or mentorship as part of a self-care intervention for anorexia nervosa. This strategy is consistent with the philosophy of the *recovery approach*, which has been embraced as national mental health policy in England [22] and Scotland [23]. According to this approach, peer support is one of the five main processes of recovery, alongside hope or optimism about the future and confidence to change, development of identity, having a sense of meaning in life, and experiencing empowerment [24]. Connecting with others with similar problems, sharing skills and choosing recovery goals are key processes within this approach [25]. The use of peer support is also advocated by self-determination theory, which posits that autonomy, self-competence and relatedness to others are the bases that drive internal motivation and growth [26]. Research in eating disorders has linked perceived autonomy support from parents, staff and treatment peers to higher motivation to change [27,28] and greater self-compassion [27], whereas low self-efficacy impairs treatment outcomes [29]. Relatedness is also gravely compromised in anorexia nervosa, with loneliness [30] and poor social relationships [31] serving as causal and maintaining factors [31]. Thus, the use of peer mentors is not only linked with increased access and availability of interventions, but also offers a theoretically grounded approach for helping individuals with anorexia nervosa.

*Recovery* MANTRA integrates two novel strategies to provide peer support to individuals with anorexia nervosa. The first strategy involves offering a library of recorded personal recovery narratives (derived from interviews with formerly ill individuals) that describe successful change steps and the nonlinear process of recovery. A feasibility and acceptability study of an early prototype of the guided self-help intervention in a community sample of participants with anorexia nervosa was promising; participants exhibited weight increases and symptom improvement subsequent to the intervention [32]. Previous test trials have demonstrated that watching similar video podcasts (‘vodcasts’) to support eating or improve positive mood lead to increases in test meal consumption and reductions in anxiety and negative thoughts [33,34]. Feedback from participants, carers, and professionals has led to improvements in these materials [18].

The second strategy to provide peer support in *Recovery* MANTRA involves weekly text-based guidance from peer mentors with personal experience of eating disorders. These online guidance sessions are designed to enhance motivation and complement the participant’s use of a self-care workbook. Preliminary evidence regarding the use of peer mentors for psychological interventions is encouraging. For instance, a Cochrane systematic review examining evidence of the effectiveness of consumer providers of care concluded that these providers were as effective as statutory mental health services [35]. Moreover, the use of peer mentors as support for people with eating disorders has been found to improve these people’s quality of life and increase adherence with services [36]. However, the quality of the guidance has been found to be an important factor in shared information approaches [5] and thus rigorous, standardized training and supervision of peer mentors is an integral component of delivering an efficacious intervention. Indeed, research suggests that training of nonspecialized professionals is an important factor in enhancing feasibility of task-sharing interventions [37]. We have previously had success in training people with experience of eating disorders (mainly as caregivers) to provide a form of motivational interview-based support to other families [38] and will therefore follow the same strategy for training peer mentors in *Recovery* MANTRA.

**Figure 1:**
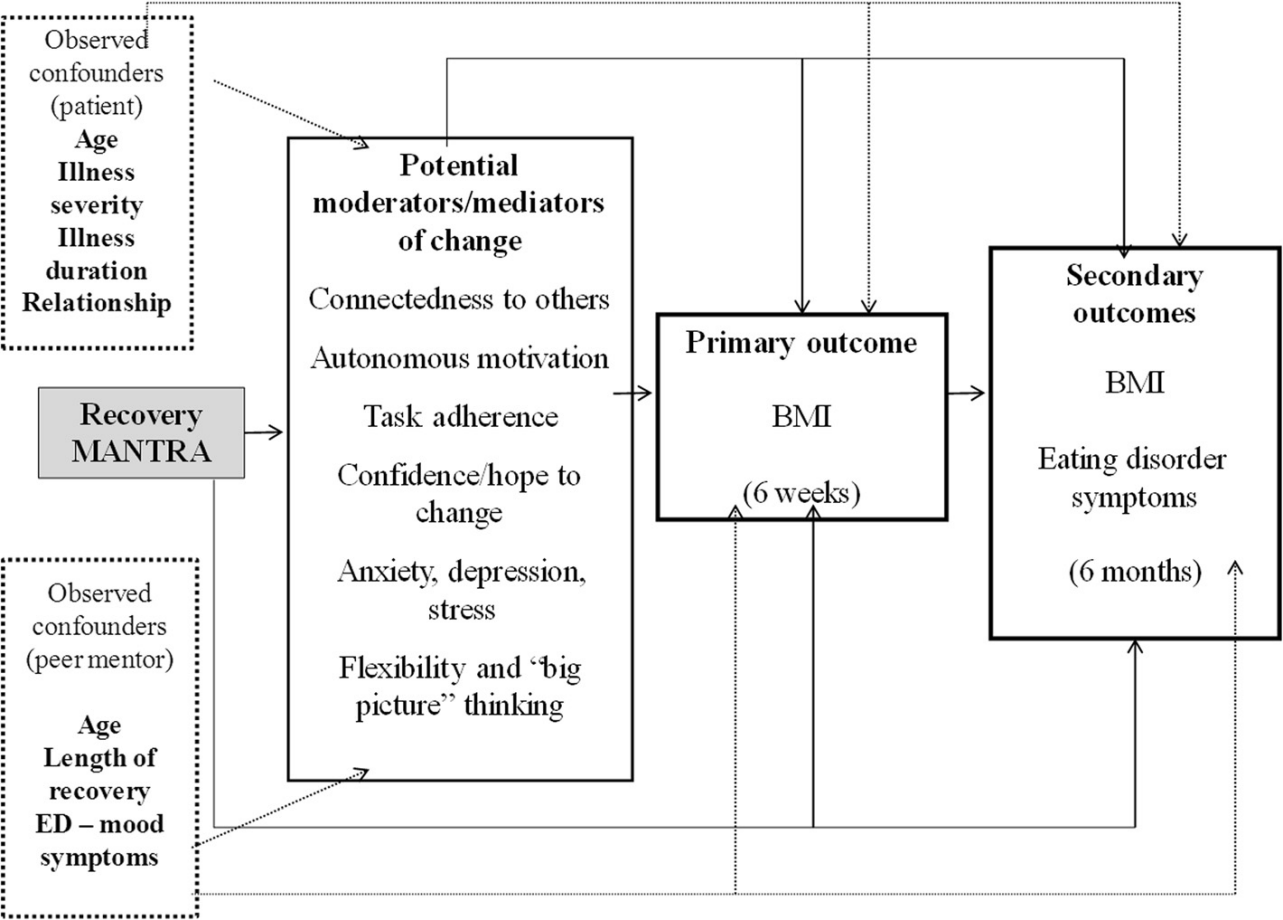
Participants’ and peer mentors’ confounders, moderators and mediators, and primary and secondary outcome measures for *Recovery* MANTRA. BMI, body mass index.

The objectives of the aforementioned strategies for peer mentorship are to inspire, illustrate and model change for individuals with anorexia nervosa. These strategies focus *Recovery* MANTRA intervention efforts on modifiable processes, such as motivation and confidence to change, adherence to treatment, social adjustment, and mood early in treatment, and may therefore enhance treatment outcomes as compared with treatment as usual. Figure 1 outlines how the elements of the *Recovery* MANTRA intervention will target the hypothesized moderators and mediators of change within our model.

### *Recovery* MANTRA: study aims and research questions

The aims of the current study are threefold: (1) to assess the impact of adjunct *Recovery* MANTRA on body mass index (BMI) and eating disorder symptoms for adults in the early stages of outpatient treatment for anorexia nervosa; (2) to assess the impact of *Recovery* MANTRA on theorized mechanisms of change (and thereby evaluate mediators and moderators of BMI and eating disorder symptom outcomes), and (3) to assess the acceptability, feasibility, and treatment fidelity for the intervention and the impact of providing guidance on peer mentors’ own BMI and eating disorder symptoms. Specifically, the primary, secondary, tertiary, and exploratory research questions are outlined as follows.

#### Primary

Is *Recovery MANTRA* associated with higher BMI at the end of the 6-week intervention relative to treatment as usual alone?

#### Secondary

Is *Recovery MANTRA* associated with higher BMI at 6-month follow-up and with lower eating disorder symptoms at 6-weeks, and 6-month follow-up compared with treatment as usual alone?

#### Tertiary

Do the domains identified by the underlying cognitive-interpersonal theoretical model (including motivation, hope, and confidence to change, anxiety, stress, and depression, perceived therapeutic alliance, work and social adjustment, social support, and increased cognitive flexibility) moderate or mediate changes in primary and secondary outcomes? Does the trajectory of daily process measures and weekly eating disorder symptoms measures predict outcomes (BMI and eating disorder symptoms) at 6-month follow-up?

#### Exploratory

1. To what extent are peer mentors able to adhere to the intervention guidelines and provide guidance or support in a theoretically supported, consistent manner? Further, what is the impact of guidance delivery on eating disorder symptoms and mood among the peer mentors?
2. How do participants and peer mentors rate the acceptability and utility of the intervention?
3. Do ratings of treatment adherence or fidelity and acceptability predict outcomes at 6-month follow-up?

## Methods

### Trial design

This is a multicentre, two-arm randomized controlled study (SHARED) exploring the efficacy of adding a guided self-help intervention (*Recovery* MANTRA) to treatment as usual for anorexia nervosa. The study received ethical approval from a National Research Ethics Service Committee (London Brent, approval number 14/LO/1347) and approvals from the research and development departments at South London and Maudsley NHS Foundation Trust, Barnet Enfield & Haringey NHS Mental Health Trust, Coventry and Warwickshire Partnership NHS Trust, Surrey and Borders Partnership NHS Foundation Trust, Worcestershire Health and Care NHS Trust, Cambridgeshire and Peterborough NHS Foundation Trust, Cumbria Partnership NHS Foundation Trust, Dorset HealthCare University NHS Foundation Trust, Leicestershire Partnership NHS Trust, Lincolnshire Partnership NHS Foundation Trust, South Essex Partnership University NHS Foundation Trust, Nottinghamshire Healthcare NHS Trust, Sussex Partnership NHS Foundation Trust and Kent and Medway NHS Trust. Recruitment will not begin at any individual centre until all approvals have been obtained.

After providing informed consent, participants are randomly assigned to either (1) *Recovery* MANTRA in addition to treatment as usual (*Recovery* MANTRA condition) or (2) treatment as usual alone (control condition). Participants are recruited from over 25 different specialist adult outpatient eating disorder services in the UK. Data are collected at baseline, during the intervention, after 6 weeks (that is, at the end of the intervention for those in the *Recovery* MANTRA condition), and at 6-month follow-up (Table 1). At the end of the study, individuals who had been randomized to the treatment as usual only control condition will be offered the *Recovery* MANTRA intervention materials (that is, vodcasts and workbook).

**Table 1:** **Schedule of assessments in** ***Recovery*** **MANTRA**

**Assessment**	**Eligibility screening**	**Baseline**	**Daily, for 6 weeks**	**Weekly, for 6 weeks**	**End of 6 weeks**	**6-month follow-up**
Participant’s information, informed consent, screening	X					
Clinical and demographic information		X				
Body mass index		X		X	X	X
Eating Disorder Examination Questionnaire		X			X	X
Depression Anxiety Stress Scale		X			X	X
Work and Social Adjustment Scale		X			X	X
The Autonomous and Controlled Motivations for Treatment Questionnaire		X			X	
Eating Disorder Examination Questionnaire - Short				X		
Daily assessments			X			
Client Service Receipt Inventory						X
Visual analogue scale: confidence and motivation to change		X			X	
Cognitive flexibility		X			X	
Patient alliance with therapist		X			X	
Patient alliance with peer mentor				X		

Daily assessments employ visual analogue scales and follow the day reconstruction method to measure daily mood, motivation, confidence, treatment adherence and connectedness.

### Randomization

Participants will be randomized by an independent researcher using stratified randomization by centre and illness severity (severe illness will be defined as BMI <16 kg/m^2^). Once the database has returned a participant’s group allocation, no changes can be made.

### Participants

Patients aged 16 or over who are referred to one of the participating specialist adult outpatient eating disorder clinics with a primary DSM-5 diagnosis of anorexia nervosa or other specified feeding or eating disorder with a BMI of 18.5 kg/m^2^ or below are offered an opportunity to participate in the research. ‘Other specified feeding or eating disorder’ will be defined as having features of anorexia nervosa but missing at least two of the four diagnostic criteria. Patients with insufficient knowledge of English, or with severe mental or physical illness needing treatment in its own right (for example, psychosis or diabetes mellitus) will be excluded. The participants will be reimbursed for their time at the completion of all assessments.

### Sample size

Previous research suggests that the effect size for increases in BMI subsequent to a guided self-help intervention is 0.29 [6]. To detect the same level of change in our primary outcome variable (BMI) using a paired-samples *t* test (*α* = 0.05, *β* = 0.20), a minimum of 90 participants would be required per study arm. However, owing to limited resources and because this is a feasibility study, we expect to recruit 75 participants per arm (resulting in total *N* = 150). This would enable us to detect an effect size of 0.33 or larger (*α* = 0.05, *β* = 0.20).

### Recruitment

After initial assessment by the clinical team, patients will be informed about the study by the local principal investigator or clinical support officer at the study sites. All participants will be clearly informed of the voluntary nature of the study.

### Treatment arms

#### Treatment as usual

The group allocated to this condition will receive treatment as usual at the participating site and will also be asked to complete assessments on the Ieso Digital Health platform. Assessments and a minimal level of feedback will occur following the same schedule as for the *Recovery* MANTRA condition (see Table 1). Treatment as usual will vary between centres (for example group psychoeducation versus individual psychotherapy or versus nutritional support and medical monitoring). These differences will be assessed and taken into account when analyzing and interpreting findings.

#### Guided self-help (Recovery MANTRA)

Participants allocated to the *Recovery* MANTRA condition will receive self-help materials and weekly guidance from a peer mentor to supplement their treatment as usual. The self-help materials include a collection of short video clips (‘vodcasts’) that describe various strategies for behaviour change, anxiety management techniques, and recovery narratives generated by individuals with personal experience of eating disorders. The vodcasts are accompanied by (and signposted in) a self-care workbook. The workbook provides supplemental information and offers the rationale for the behaviour change steps proposed. The intervention materials were developed with the aim of increasing confidence to change, internalized motivation, connectedness to others and hope. In addition, the participants will be offered individualized support via online text chat (using a secure platform) through peer mentors for a maximum of one hour each week over 6 weeks. The peer mentor will contact the participants to introduce the materials and to provide information about the parameters of support (see Table 2 for programme of guidance).

**Table 2:** **Programme of guidance for participants from peer mentors**

*Session 1: Orientation, motivation and preparation*	Aim: introduce intervention and increase perceived value of making changes.
	Elicit participant view and increase motivation by reinforcing change talk. Instruction on use of Ieso Digital Health platform, vodcasts, APT strategies (that is, reflection tasks, implementation intentions, behavioural experiments). Review individual priorities to determine the order for proceeding through the four core workbook chapters.
*Session 2: Cognitive strengths and challenges*	Aim: increase flexibility, spontaneity and bigger picture thinking by following APT structure.
*Session 3: Social strengths and challenges*	Aim: support making changes in social context and identifying family relationships and healing ruptures.
	Follow APT process to enlist social support through friends, family, and work colleagues.
*Session 4: Nutrition*	Aim: review strategies for reducing avoidance behaviours (such as restriction, vomiting, or exercise).
	Follow APT with planning and anticipating obstacles, such as stress or abdominal discomfort.
*Session 5: Emotional strengths and challenges*	Aim: review strategies for increased emotional expression toward others (for example, through writing, openness), reducing maladaptive emotional regulation, enhancing self-compassion and nurturance.
*Session 6: Consolidation and re-setting goals*	Aim: exchange of ‘goodbye letters’.
	Offer a summary of progress and suggestions for further goals. Develop a set of personal resources (for example, vodcasts, written materials) to maintain change over time.

APT, ‘awareness, planning, try it’.

The workbook is organized into six sections: introduction to the intervention; separate modules detailing cognitive, emotional, social or interpersonal, and nutritional factors; and a summary section for future planning and integration of content. Each section follows a similar format and includes ‘*awareness*, *planning* and *try it*’ (APT) sections. *Awareness* includes psychoeducation and reflective exercises. *Precise planning* includes implementation of intention strategies for specific, foreseeable obstacles, in order to keep the overall goal of change in mind (in the form of ‘if… then’ statements) and using daily living prompts as hooks for new habits. *Try it* exercises include small behavioural experiments that encourage patients to take small, manageable risks in challenging thoughts, behaviours, and patterns related to eating disorders as they work toward recovery. The workbook is therefore customizable to meet individual needs; the chapters are allocated one to a week but may be utilized in whichever order participants see fit (except for the introductory and concluding chapters), based on level of confidence and sense of priority. The introduction section includes exercises focused on evaluating and increasing readiness to recover and identifying priorities and reasons for change, followed by APT goals setting. The summary section includes exercises for reflecting on the skills learned in the previous sections, the progress that has been made and re-evaluating the reasons for and one’s confidence to change. Each of the cognitive, emotional, social and nutritional modules target the difficulties a patient might experience in the respective area through various engaging exercises (for example, *cognitive module*: practising comprehensive, flexible thinking; *emotional module*: attending to positive emotions and building self-compassion; *social and interpersonal module*: strengthening relationships; *nutritional module*: confronting avoidance and managing cravings and purgative impulses; see Table 2).

A total of 60 vodcasts are downloadable from a secure website (Ieso Digital Health; http://www.iesohealth.com/) and participants are encouraged to save these locally on internet-capable mobile devices, such as iPods, iPhones, or Android phones. Consistent with the organization of the accompanying workbook, vodcasts are organized into six categories (that is, one set of vodcasts for each workbook section), and each vodcast lasts approximately 90 to 180 seconds. The ‘recovery narrative’ vodcasts were recorded via semistructured interviews conducted with recovered patients as they described what was helpful for their recovery, what they wished they had known at the time of their illness, and their descriptions of the nature of their recovery experiences. Nondistracting, soothing animations were added to complement the recovery narratives. Finally, each vodcast ends with a brief, written prompt that is directly linked to the content of that vodcast as a way to promote additional reflection, planning and active behaviour change.

In addition to the recovery narratives, the vodcast directory includes ‘skills-sharing’ vodcasts that are focused on helping patients gain specific skills for changing eating disorder behaviours (for example, managing food cravings, mindful eating, managing distressing emotions, focusing on the bigger picture and flexible thinking, and building self-compassion). Participants are also advised to download their own relaxation, mindfulness, and distraction podcasts in addition to those provided within the collection of vodcasts.

In total, 10 to 15 peer mentors will be trained, to allow for attrition. The purpose of the guidance is not to provide an additional stand-alone intervention, but rather, to offer support to patients in using the self-help materials effectively and purposefully. Peer mentors are individuals with past experience of eating disorders who have sufficient time for training, supervision and coaching and are trained in professions allied to medicine or equivalent (psychology, counselling) or teaching. Individuals with clinically significant levels of baseline anxiety, depression or eating disorder symptoms are excluded. Comprehensive training for task-sharing interventions involves three important components: participation in an introductory workshop delivered by an expert, reading or review of workbooks or other written materials that detail the intervention, and administration of the intervention under close supervision of treatment experts [2]. Peer mentors will receive all of these training components prior to delivering the *Recovery* MANTRA intervention.

The curriculum currently used for training and supervision of carer coaches will be adapted for training peer mentors. In particular, the peer mentors will receive three days of training in motivational interviewing, during which they will also become familiarized with the *Recovery* MANTRA materials and practise using the online text-chat delivery system. For the duration of their involvement in the project, the peer mentors will also receive weekly clinical supervision and will undergo regular assessment of their eating disorder symptoms and mood. Copies of the text-chat messages between the peer mentor and the participant will be used for supervision and to assess treatment quality and fidelity.

### Fidelity and adherence to model

Peer mentors will be assigned study participants only after they have demonstrated sufficient competence with training samples (that is, the training samples will have details of patients who will not be used within the study). Adherence and fidelity to the motivational interviewing model will be monitored by an analysis of a random selection of transcripts (sessions 2 to 5) using the Motivational Interviewing Treatment Integrity rating system, a validated and reliable behavioural coding system used to measure treatment integrity in motivational interviewing [38]. A simplified version of the Revised Cognitive Therapy Scale [39] will be adapted specifically for this intervention to address the more goal-driven aspects of the programme.

### Patient assessments

The assessments will be conducted at different time points (that is, baseline, daily, 6-weeks, and 6-month follow-up; see Table 1 for the assessment schedule). Patients will complete the self-report measures on the Ieso Digital Health website (http://www.iesohealth.com/).

The following self-report measures will be used in the assessment:

1. Demographic questionnaire: created by the study authors.
2. Eating Disorder Examination Questionnaire (EDE-Q; [40]): 36-item self-report measure to assess eating disorder symptoms. The EDE-Q has high internal consistency [41] and moderate to high concurrent and criterion validity [42].
3. Eating Disorder Examination Questionnaire Short (EDE-QS; Gideon et al., unpublished measure): 12-item self-report measure of eating disorder symptoms derived from the EDE-Q.
4. Depression, Anxiety and Stress Scales (DASS-21; [43]): 21-item self-report measure of depression, anxiety and stress. The scale has been validated and found to possess good reliability with Cronbach’s *α* to be 0.94 for depression, 0.87 for anxiety and 0.91 for stress [44].
5. Work and Social Adjustment Scale [45]: 5-item, reliable and valid measure of patients’ perceptions of impairment in everyday functioning. The internal scale consistency ranged from 0.70 to 0.94 and the test-retest correlation was 0.73 in the validation study [45].
6. The Autonomous and Controlled Motivations for Treatment Questionnaire (ACMTQ; [46]): 12-item self-report questionnaire adapted from the Treatment Self-Regulation Questionnaire designed to measure aspects of motivation [46]. Previous research suggests adequate internal consistency in a clinical sample of people with depression (Cronbach’s *α* was 0.77 for autonomous motivation and controlled motivation scores in the original psychometric study [46]. A principal components analysis with an eating disorder sample reported two distinct factors, accounting for 28.66% and 24.05% of the variance, respectively, with Cronbach’s *α* of 0.78 for both subscales [47].
7. Visual analogue scales: importance and confidence to change: Participants will be asked to rate how important it is for them to change (from 0 for ‘not at all important’ to 10 for ‘extremely important’) and how confident they are in their ability to change (from 0 for ‘not at all confident’ to 10 ‘extremely confident’).
8. Cognitive flexibility: 4-item self-report questionnaire created by the study authors to assess the extent to which participants attend to small details versus the ‘big picture’, that is, their tendencies to stick to rules and engage in rituals versus their spontaneity.
9. Perceived alliance with therapist: 5-item self-report questionnaire created by the study authors to assess the extent to which participants feel understood by their therapists, experience confidence in their therapists, believe they are working toward mutually agreed-upon goals with their therapists, trust their therapists, and feel that their therapists offers new ways of looking at their problems.
10. Perceived alliance with peer mentor (created by the study authors and to be administered only to the Recovery MANTRA group): two questions will be administered weekly during the 6-week intervention phase to assess comfort (*How often do you feel comfortable working with your peer mentor*) and alliance (*How often do you and your peer mentor agree on what needs to be done to improve your situation*).
11. Ultra-brief daily surveys (created by the study authors of this manuscript): 9 items measuring treatment motivation, hope, mood, treatment compliance, and feelings of connection to others. Items will be completed at the end of each day during the 6-week intervention period. Day reconstruction method procedures will be employed [48] to enhance the reliability of end-of-day mood reports [49].

Patients’ weights will be recorded at baseline and weekly for 6 weeks, by clinicians. Patients will also be asked to self-report their BMI at 6-month follow-up (in previous studies, we have found high correlations between clinician and patient ratings; Rhind *et al.*, unpublished observations).

### Peer mentor assessments

Peer mentors will complete a demographic questionnaire upon enrolment and the EDE-QS and DASS-21 at baseline and every 3 months for the duration of their involvement in the project.

### Treatment as usual assessment

Treatment as usual will vary between centres and this will impact the effect size of the intervention [50]. We will therefore conduct structured interviews with clinicians at each study site to clarify the nature of treatment as usual at each centre. We will also use an adapted form of the Client Service Receipt Inventory (a well-established tool used in health economics) to track the amount of treatment received over the year. The inventory will be administered at 6-week and 6-month, to measure patients’ service usage.

### Outcome assessment

The primary outcome variable is patient BMI at the end of the 6-week intervention (treatment as usual versus *Recovery* MANTRA). The secondary outcome variables are patient BMI at 6-month and follow-up and eating disorder symptoms (assessed by EDE-Q scores) at 6-week and 6-month follow-up (treatment as usual versus *Recovery* MANTRA). The hypothesized mediators and moderators of primary and secondary outcomes include mood (DASS-21), perceived work or social adjustment (Work and Social Adjustment Scale), motivation and confidence to change (visual analogue scale and ACMTQ), cognitive flexibility, and perceived alliance with therapist and peer mentor. Moreover, the trajectories of change in eating disorder symptoms (weekly EDE-QS), perceived alliance with peer mentors (assessed weekly), and mood, confidence or motivation, connection to others, hope and adherence or satisfaction with treatment plan (assessed daily via ultra-brief surveys) will predict the primary and secondary outcomes. Exploratory outcomes include the impact of guidance delivery on peer mentors’ eating disorder symptoms and mood (assessed via a feedback form) and treatment adherence or fidelity and acceptability (assessed via a feedback form) on secondary outcomes.

## Expected results

### Quantitative data analysis

This exploratory hypothesis testing study is designed to assess our theorized model of maintenance and change in anorexia nervosa (Figure 1). The time trend in weight and symptoms of anorexia nervosa over 6 months for each person will be estimated using mixed-effects regression models with structured covariance patterns to account for autocorrelation of repeated measures [51,52]. Models will test for linear and curvilinear change over time. The difference between the groups in the rate of change over time will then be examined using group-by-time interaction. Variation within individuals will be modelled as ‘Level 1’ and will include daily survey data, and variation between individuals (including the intercept and slope) will be modelled as ‘Level 2’. All continuous variables will be grand mean centred and results will be modelled using random intercept and slopes. Separate analyses will regress (1) participants’ weight change (baseline to 6-month follow-up) on mood, (2) participants’ weight change (baseline to 6-month follow-up) on motivation to change and on confidence to change and (3) treatment condition on repeated assessment of weight.

Moderator and mediator analyses will be performed using the regression-based bootstrapping approach described by Hayes [53]. Bias corrected bootstrap confidence intervals will be used to test moderators and mediators based upon 10,000 bootstrap samples. Analyses will be conducted using the PROCESS macro [54].

### Subsidiary analysis of peer mentors

To assess interrater reliability of the competency measure, intraclass correlation coefficients will be estimated using a one-way analysis of variance (ANOVA) model for each of the measure’s components. Any changes in symptoms among the peer mentors (for example, mood, eating) will be assessed.

### Qualitative data analysis

Three qualitative studies will be conducted.

#### Qualitative studies A and B

Participants (*n* = 75) and peer mentors (*n* = 10 to 15) will be asked to complete a feedback form at the end of the intervention. Questions will focus on overall experience of participation, use and acceptability of materials, relationship with peer mentor or participant, any particular learning points, challenges met, and suggestions for improvement. A thematic analysis will be used to analyze data from the written feedback forms [55].

#### Qualitative study C

The key themes of narratives from peer mentors (*n* = 10 to 15) reflecting on their experience of *Recovery* MANTRA will be analyzed using interpretative phenomenological analysis [56]. The analysis package Nvivo will be used to manage and organize the data [57].

## Discussion

The current study investigates the efficacy of the *Recovery* MANTRA intervention in improving outcomes for outpatients with anorexia nervosa while examining the salience of theorized mechanisms of action, including, motivation, hope and confidence to change, anxiety, stress, and depression, perceived therapeutic alliance, work and social adjustment, social support and increased flexibility. *Recovery* MANTRA has been designed to be affordable and scalable, with the potential for a wide reach. The process of therapy has been informed by the recovery approach in that people with personal experience of eating disorders are recruited as peer mentors and ‘vodcast’ narratives from people who have recovered are used to describe strategies to implement change. We are using new technologies, such as computerized assessment and feedback, live text-chat support and prompts to change that are accessible by mobile phones.

Although there has recently been a proliferation of mobile applications designed to assist with behaviour change among those with eating-related difficulties, there has been inadequate discussion of the practical, ethical and conceptual considerations [18] and a suggestion that extant eating disorder intervention applications minimally follow evidence-based principles for treatment and fail to capitalize on the unique features of mobiles, such as opportunities for repeated self-assessment [58]. Thus, assessment of a theoretically grounded and novel mobile-delivered intervention such as *Recovery* MANTRA could make a significant contribution to the limited evidence base for the use of technology to support recovery from eating disorders.

### Limitations and challenges

This will be a pragmatic trial and will examine the impact of the addition of the *Recovery* MANTRA intervention to treatment as usual in a variety of treatment settings. Although it might be difficult to capture the exact nature of treatment as usual in each centre, our strategy of randomization stratified by centre will minimize any bias that might be caused by site-related differences. A key advantage of the current design is that we can examine how feasible and acceptable this intervention is in standard treatment settings. Although we have previously used peer support in the context of interventions for carers [38] and frequently employ such individuals in our inpatient setting to help with teaching and various aspects of the therapeutic environment, we have not yet employed recovered individuals exactly in the capacity as in the *Recovery* MANTRA intervention. We will thus examine whether this form of task-sharing is feasible. We will also pay close attention to the well-being of these individuals and use their feedback to inform this recovery approach strategy. Notably, our use of audio recordings from recovered individuals as therapeutic prompts maximizes the power of the recovery approach and self-determination theory. Following our pilot testing of these recovery narratives, we decided to anonymize the vodcasts by substituting the images of the narrators with secondary images. This strategy reduced the resources required to disseminate the materials, retaining their appeal due to authenticity while allowing them to be more generally distributed without privacy concerns. Finally, the technology platform that we are using (Ieso Digital Health) has been successfully used to deliver cognitive behaviour therapy in the context of IAPT (Improving Access to Psychological Therapies) services in the UK [59], but we are not aware of this type of approach being used for people with eating disorders as yet.

## Trial status

The study started in November 2014. We are currently obtaining local research and development approvals from the participating sites. Data collection started in March 2015 at those sites that received local research and development approval.

## Abbreviations

ACMTQ: The Autonomous and Controlled Motivations for Treatment Questionnaire
ANOVA: analysis of variance
APT: ‘awareness, planning, try it’
BMI: body mass index
DASS-21: 21-item Depression Anxiety and Stress Scales
EDE-Q: Eating Disorder Examination Questionnaire
EDE-QS: Eating Disorder Examination Questionnaire - Short
IAPT: Improving Access to Psychological Therapies
MANTRA: Maudsley Model of Treatment for Adults with Anorexia Nervosa
SHARED: Self-Help and Recovery Guide for Eating Disorders
